# ATP-Mediated Transactivation of the Epidermal Growth Factor Receptor in Airway Epithelial Cells Involves DUOX1-Dependent Oxidation of Src and ADAM17

**DOI:** 10.1371/journal.pone.0054391

**Published:** 2013-01-18

**Authors:** Derek Sham, Umadevi V. Wesley, Milena Hristova, Albert van der Vliet

**Affiliations:** 1 Department of Pathology, College of Medicine, University of Vermont, Burlington, Vermont, United States of America; 2 Department of Microbiology and Molecular Genetics, College of Medicine, University of Vermont, Burlington, Vermont, United States of America; 3 Vermont Lung Center, University of Vermont, Burlington, Vermont, United States of America; University of Illinois at Chicago, United States of America

## Abstract

The respiratory epithelium is subject to continuous environmental stress and its responses to injury or infection are largely mediated by transactivation of the epidermal growth factor receptor (EGFR) and downstream signaling cascades. Based on previous studies indicating involvement of ATP-dependent activation of the NADPH oxidase homolog DUOX1 in epithelial wound responses, the present studies were performed to elucidate the mechanisms by which DUOX1-derived H_2_O_2_ participates in ATP-dependent redox signaling and EGFR transactivation. ATP-mediated EGFR transactivation in airway epithelial cells was found to involve purinergic P2Y_2_ receptor stimulation, and both ligand-dependent mechanisms as well as ligand-independent EGFR activation by the non-receptor tyrosine kinase Src. Activation of Src was also essential for ATP-dependent activation of the sheddase ADAM17, which is responsible for liberation and activation of EGFR ligands. Activation of P2Y_2_R results in recruitment of Src and DUOX1 into a signaling complex, and transient siRNA silencing or stable shRNA transfection established a critical role for DUOX1 in ATP-dependent activation of Src, ADAM17, EGFR, and downstream wound responses. Using thiol-specific biotin labeling strategies, we determined that ATP-dependent EGFR transactivation was associated with DUOX1-dependent oxidation of cysteine residues within Src as well as ADAM17. In aggregate, our findings demonstrate that DUOX1 plays a central role in overall epithelial defense responses to infection or injury, by mediating oxidative activation of Src and ADAM17 in response to ATP-dependent P2Y_2_R activation as a proximal step in EGFR transactivation and downstream signaling.

## Introduction

The respiratory epithelium forms a first line defense against inhaled pathogens and pollutants, and has developed intricate innate response mechanisms against diverse environmental challenges to provide important initial host defense and to protect airway structure and function. Many recent lines of evidence indicate that airway epithelial surface signaling through the epidermal growth factor (EGFR) represents a common pathway in many such innate host responses, and plays a key role in several protective epithelial responses to a range of environmental triggers [1], [2], [3]. EGFR is the prototypical member of the ErbB family, which comprises four receptors (HER1/EGFR/Erb1, HER2/Neu/Erb2, HER3/Erb3, and HER4/Erb4), of which EGFR, Erb2 and Erb3 are expressed within human airway epithelia. Activation of ErbB receptors by their cognate ligands results in receptor homo- or heterodimerization leading to (auto)phosphorylation within the intrinsic kinase domain and activation of downstream signaling. However, EGFR activation in response various diverse environmental or microbial stresses typically involves the initial stimulation of various G-protein-coupled receptors (GPCR), which promotes EGFR transactivation by as yet incompletely understood mechanisms involving ligand-independent intracellular mechanisms as well as activation of EGFR ligands by ADAM (a disintegrin and metalloproteinase) family sheddases [4], [5], [6], [7]. One GPCR family of particular interest in the context of epithelial injury and wound responses includes purinergic receptors, which are activated by epithelial release of ATP in response to both mechanical and molecular stresses [8], [9], and are critical in epithelial responses to injury or infection promoting mucociliary clearance and stimulating cellular repair mechanisms [8], [10], [11], [12], and transactivation of EGFR has been implicated in these ATP-mediated wound responses in various cell systems [13], [14], [15].

The mechanisms by which GPCR stimulation results in EGFR transactivation are diverse and incompletely understood, but a number of reports implicate the contribution of regulated production of H_2_O_2_ [16], [17], [18]. Proposed mechanisms in H_2_O_2_-dependent EGFR activation include oxidative inactivation of protein tyrosine phosphatase 1B to augment and prolong EGFR [16], [17], as well as oxidative modification of EGFR itself in response to ligand stimulation [19]. Moreover, H_2_O_2_ or related ROS are also thought to contribute to ADAM17 activation by ATP or other stimuli, although the oxidative mechanisms of ADAM17 activation are unclear and have been suggested to involve oxidative cysteine switch activation of pro-ADAM17 at the epithelial cell surface [20], although this has been questioned [21], [22], [23], . Alternatively, ADAM17 activity may be controlled by oxidative disulfide bonding within the extracellular domain of the mature enzyme [25], [26], although its relevance for ATP-mediated EGFR activation is unclear. Another potential mechanism by which H_2_O_2_ may mediate EGFR transactivation is by oxidative activation of non-receptor tyrosine kinases of the Src family [27], [28], which promote EGFR phosphorylation at selected residues in a ligand-independent fashion [29], [30]. The activity of Src is tightly controlled by inhibitory tyrosine phosphorylation at Y527 and by auto-phosphorylation at Y416 during activation, but recent evidence indicate that Src family kinases are also regulated by oxidation of conserved cysteine residues with the C-terminal region [31], [32], [33], and such oxidative modification of Src kinases have been implicated in cell adhesion and spreading and in wound responses [31], [34].

The oxidative mechanisms involved in EGFR activation also critically depend on the origin of H_2_O_2_ production. While some studies have implicated mitochondria-derived H_2_O_2_ or related reactive oxygen species (ROS) in ATP-mediated EGFR activation [18], ATP-dependent production of H_2_O_2_ by airway epithelial cells originates primarily from the NADPH oxidase homologs DUOX1 and DUOX2, which are prominently expressed in the airway epithelium [24], [35]. Indeed, DUOX1 has been implicated in epithelial EGFR activation in response to various stimuli including ATP [24], [36], although the mechanisms are incompletely understood. The present studies were designed to detail the mechanisms by which extracellular ATP promotes transactivation of EGFR within airway epithelial cells, and to establish the involvement of DUOX1/2 as major H_2_O_2_ sources within the respiratory epithelium. Our results demonstrate that ATP-dependent EGFR transactivation within airway epithelial cells involves DUOX1-dependent activation of Src and ADAM17 by cysteine oxidation within these enzymes, as initial events in EGFR phosphorylation subsequent epithelial wound responses.

## Results

### Extracellular ATP Promotes Epithelial Wound Responses by P2Y_2_R-mediated Activation of EGFR and Src

**Figure 1:**
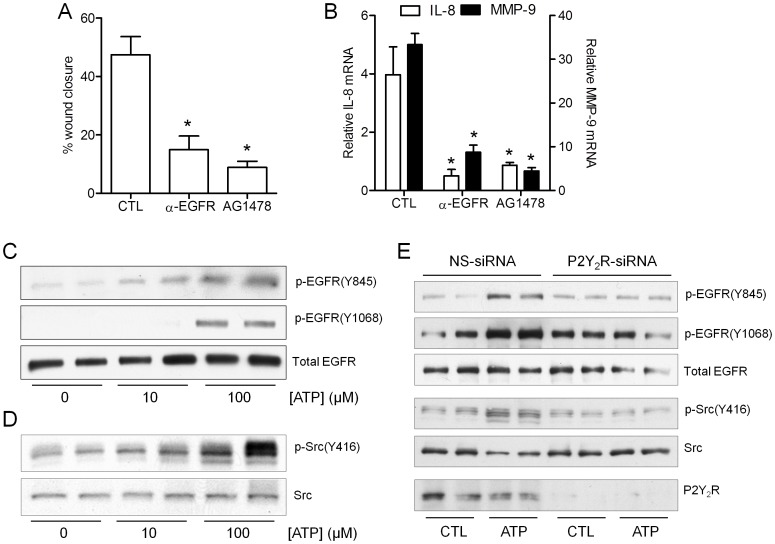
EGFR-dependent wound responses are mediated by ATP-dependent P2Y_2_R activation. Confluent H292 cells were serum-starved overnight and subjected to scratch wounding or stimulated with exogenous ATP (100 µM) for 8 hrs, and wound closure (A) or fold increases in mRNA expression of MMP-9 or IL-8 compared to non-stimulated cells (B) were determined. *: p<0.05 compared to corresponding control (n = 4). (C,D) H292 cells were stimulated with ATP for 10 min, and whole cell lysates were analyzed by Western blot using phospho-specific antibodies p-EGFR (Y845 or 1068) and unphosphorylated (total) EGFR or with antibodies against p-Src (Y416) and total Src. (E) H292 cells were pre-incubated with P2Y_2_R siRNA or control non-specific (NS) siRNA for 72 hrs, and stimulated with ATP (100 µM, 10 min) for analysis of p-EGFR, p-Src, or P2Y_2_R by Western blot. Representative results of 2 independent experiments are shown.

We first confirmed previous findings indicating a role for EGFR in epithelial wound responses (refs), using a blocking α-EGFR antibody (mAb 225; Calbiochem) or the EGFR tyrosine kinase inhibitor AG1478. Both approaches significantly inhibited epithelial wound closure in H292 cells in a model of scratch injury (**Fig. 1A**), and suppressed ATP-dependent induction of MMP-9 or IL-8 (**Fig. 1B**), established mediators of epithelial wound repair [1], [37]. Analysis of tyrosine phosphorylation within EGFR using phospho-specific antibodies showed that stimulation of H292 cells with ATP resulted in dose-dependent phosphorylation at tyrosine 1068 (Y1068), a known autophosphorylation site after ligand-dependent EGFR dimerization, and at Y845, a target for non-receptor tyrosine kinases such as Src [28], [30]. Both phosphorylation events were dose-dependently increased by ATP up to 100 µM (**Fig. 1C**), suggesting the involvement of purinergic receptor stimulation. EGFR phosphorylation at Y845 implies the involvement of Src, which was confirmed by assessing tyrosine auto-phosphorylation at Y416, which indicated that ATP dose-dependently enhanced Src phosphorylation at Y416 (**Fig. 1D**), whereas phosphorylation at Y527, which is associated with inhibition of Src, was not significantly affected by ATP (not shown).

Among the major purinergic P2Y receptors expressed in the airway epithelial cells [8] as well as H292 cells, the P2Y_2_R subtype appeared to be the most important in ATP-dependent EGFR activation, based on similar EGFR activation by the P2Y_2_R ligand UTP, but not by ADP or UDP, which primarily activate P2Y_1_R or P2Y_6_R (**Fig. S1**). Using siRNA silencing of P2Y_2_R, we confirmed its critical importance in ATP-dependent activation of EGFR and Src (**Fig. 1E**), in agreement with previous studies with other cell types 13,38,39.

### ATP-mediated EGFR Phosphorylation Depends on EGFR Ligand Binding As Well As Src Activation

**Figure 2:**
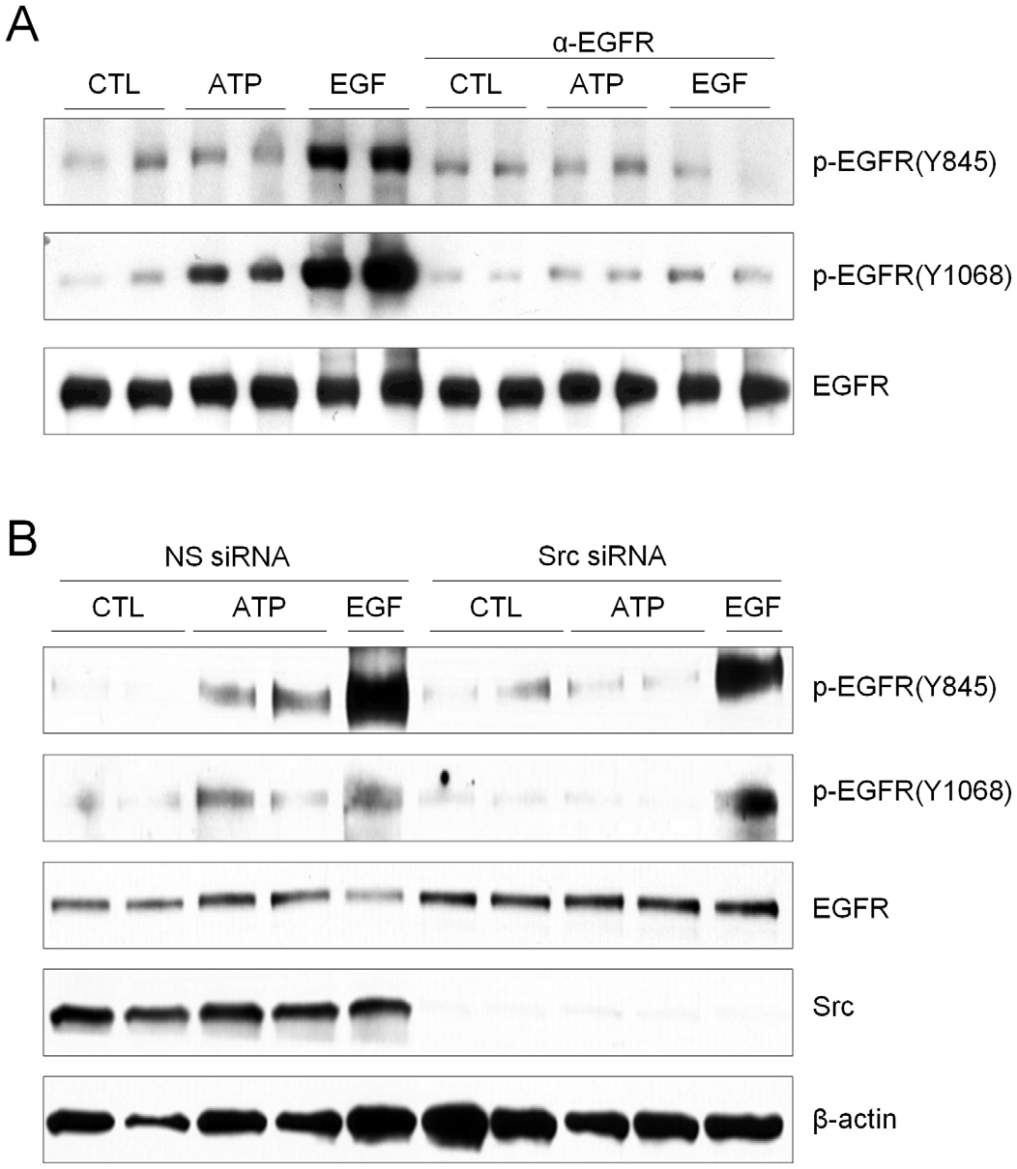
ATP-mediated EGFR phosphorylation is both ligand- and Src-dependent. (A) Confluent H292 cells were serum-starved overnight and pre-treated with α-EGFR mAb (4 µg/ml) for 30 min prior to treatment with ATP (100 µM) or EGF (100 ng/ml) for 10 min, and lysates were analyzed by Western blot using p-EGFR and total EGFR antibodies. (B) H292 cells were pre-incubated with c-Src siRNA or control non-specific (NS) siRNA for 72 hrs, and subsequently serum-starved overnight and stimulated with ATP or EGF, and cell lysates were analyzed for phosphorylated EGFR and total EGFR, c-Src and β-actin. Representative blots from 2–3 separate experiments are shown.

A range of studies have demonstrated that EGFR transactivation by GPCR is mediated by ADAM-dependent shedding of EGFR ligands and ligand-dependent EGFR dimerization and activation [4], and our previous studies demonstrated that exogenous ATP is capable of promoting shedding of the EGFR ligand TGF-α [24]. The involvement of EGFR ligand binding in EGFR transactivation by ATP was evaluated used a blocking α-EGFR antibody, which was found to almost completely prevent EGFR phosphorylation at both Y845 and Y1068, by EGF as well as ATP (**Fig. 2A**), while similar incubation with control IgG1 had no effect (not shown). ATP-mediated EGFR phosphorylation at both Y845 and Y1068 was also abrogated by the EGFR kinase inhibitor, AG1478 (10 µM), as expected (**Fig. S2A**).

Since ATP-mediated EGFR phosphorylation at Y845 and Y1068 was associated with activation of Src, we postulated that EGFR transactivation by ATP also involves Src in a ligand-independent mechanism. Indeed, ATP-mediated EGFR phosphorylation of Y845 as well as Y1068 was almost completely prevented in the presence of the pharmacological Src inhibitor PP2 (1 µM) (**Fig. S2B**) or after siRNA-dependent silencing of Src (**Fig. 2B**). In contrast, blockade or silencing of Src only suppressed EGF-induced EGFR phosphorylation at Y845 but did not affect EGFR autophosphorylation at Y1068 by EGF, indicating that ligand-dependent EGFR phosphorylation at Y845 depends on Src whereas autophosphorylation at other residues such as Y1068 is Src-independent. Collectively, these findings highlight the importance of Src activation as well as EGFR ligand shedding in EGFR transactivation in airway epithelial cells in response to extracellular ATP.

### Extracellular ATP Activates ADAM17 via Src As Well As Ligand-dependent EGFR Stimulation

**Figure 3:**
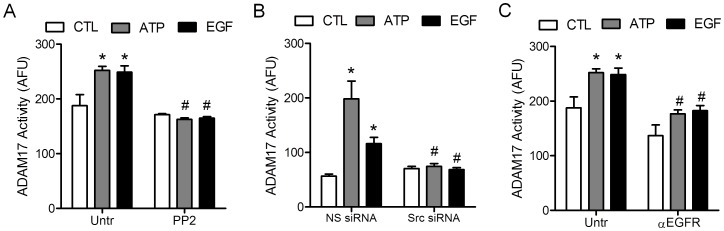
Exogenous ATP results in activation of ADAM17 via c-Src and EGFR ligand. H292 cells were preincubated with PP2 (A), Src-specific or control siRNA (B), or α-EGFR mAb (C) as in Figs 2 and 3, and stimulated with ATP (100 µM) or EGF (100 ng/ml) in the presence of a fluorogenic substrate specific for ADAM17, and changes in fluorescence were monitored after 2 hrs. *: p<0.05 compared to untreated control or NS siRNA; ^#^:p<0.05 compared with corresponding stimulation in the absence of PP2, Src siRNA, or α-EGFR mAb (n = 4).

Based on reported involvement of ADAM17 in airway epithelial EGFR activation in response to many stimuli [24], [40], we monitored ADAM17 activation using a fluorogenic ADAM17 substrate (Calbiochem). Indeed, stimulation of H292 cells with ATP enhanced fluorescence, which was prevented after siRNA silencing (**Fig. S3**), indicating that this fluorescence increase reflects activation of ADAM17. Activation of ADAM17 was observed in response to ATP as well as EGF, and was in both cases completely prevented in the presence of the Src inhibitor PP2, or after siRNA silencing of Src (**Fig. 3A,B**). Intriguingly, ADAM17 activation was also partially suppressed in the presence of α-EGFR (**Fig. 3C**), suggesting that ligand-dependent EGFR activation contributes to ADAM17 activation, possibly representing a positive feed-forward loop resulting in enhanced shedding of EGFR-activating ligands. The complete dependency of ADAM17 activation by either ATP or EGF on the presence of Src is in agreement with a recent report indicating the importance of Src in paracrine signaling of EGFR and ERK through ADAM17-mediated ligand shedding [**41**]. These collective findings indicate that EGFR transactivation by ATP is critically dependent on initial activation of Src, which in turn phosphorylates EGFR at Y845 and activates ADAM17 to promote ligand-dependent EGFR autophosphorylation.

### ATP-dependent EGFR Stimulation Occurs at the Apical Surface in Polarized Epithelia

**Figure 4:**
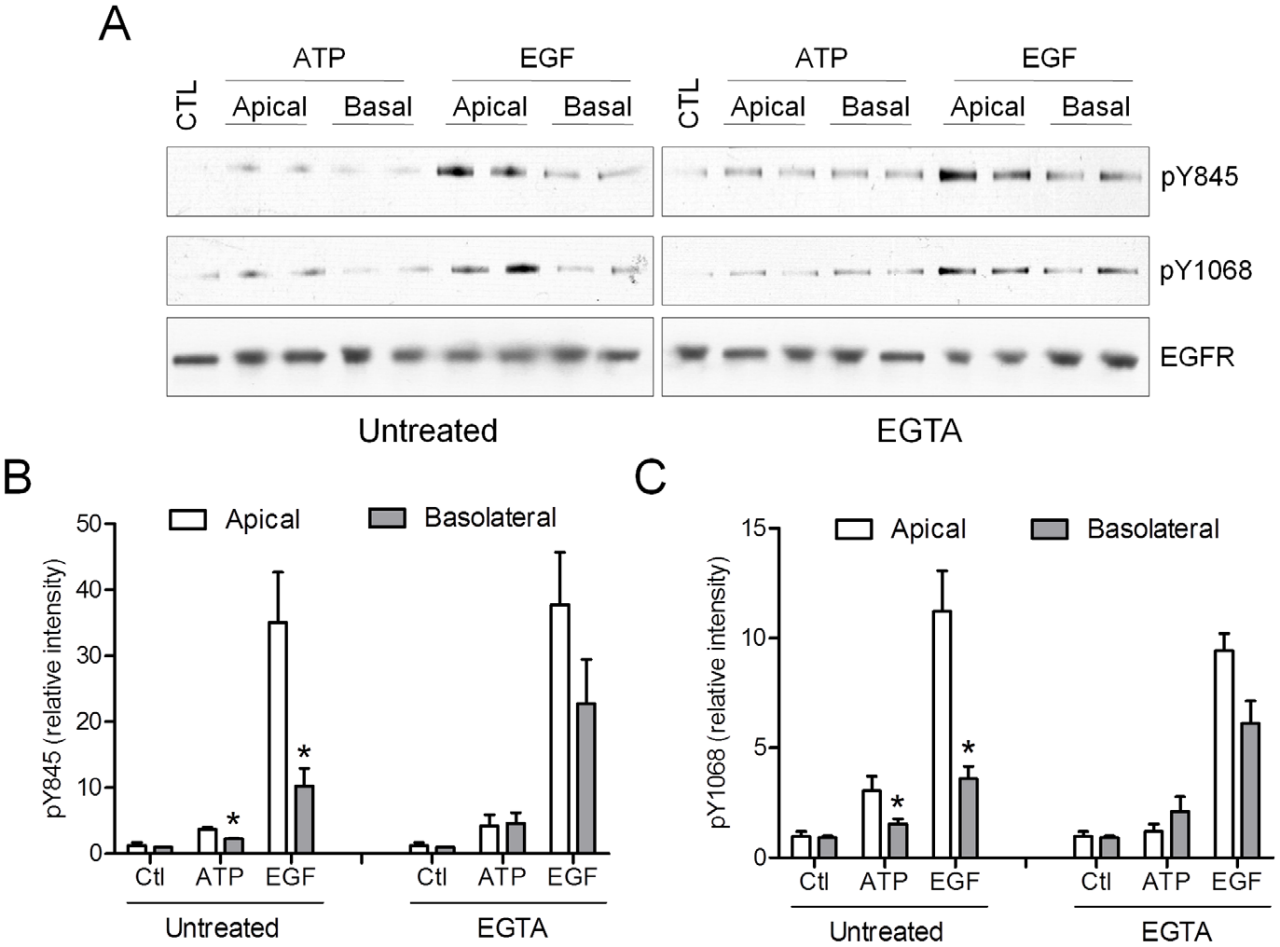
Apical ATP activates EGFR in intact polarized epithelium. Immortalized HBE1 cells were cultured in Transwell inserts until they formed a functional polarized barrier (TER >150 Ω.cm^2^) and stimulated with ATP or EGF, which was administered either apically or basolaterally. Similar stimulations were performed in cell monolayers that were pretreated with EGTA (1 mM) to disrupt tight junctions and TER. Cell lysates were prepared after 10 min and analyzed for (phosphorylated) EGFR (A). Western blots from 2–3 separate experiments were quantified by densitometry (B,C). *: p<0.05 compared to corresponding apical stimulation (n = 4).

Our findings so far were based on studies in non-polarized submerged cultures of H292 cells, which may not adequately reflect EGFR-dependent signaling in normal polarized airway epithelia, in which apically generated EGFR ligands are thought to be segregated from their receptor(s) at the basolateral membrane by epithelial junctions, allowing effective EGFR activation only under conditions of epithelial injury [42], [43]. Since ATP release upon epithelial stimulation by diverse environmental or microbial stresses occurs primarily at the apical surface, where P2Y_2_ receptors are largely localized [8], [9], we questioned whether such apically released ATP is capable of transactivating EGFR even in intact polarized epithelia. To test this, we cultured immortalized human bronchial epithelial (HBE1) cells on culture inserts at air-liquid interface, to generate polarized epithelial monolayers, and stimulated these with ATP or EGF either apically or basolaterally. As shown in **Fig. 4**, apical stimulation of polarized HBE1 cells with ATP or EGF resulted in enhanced EGFR phosphorylation at Y845 and Y1068, while such responses were significantly less pronounced upon basolateral stimulation. These differences in responsiveness were largely abolished after preincubation of polarized HBE1 cells with EGTA, which disrupts epithelial tight junctions and facilitate diffusion of extracellular mediators between apical and basolateral compartments [43]. These findings indicate that ATP is capable of promoting EGFR transactivation in intact epithelia, and does not necessarily require epithelial injury or disruption of tight junctions. Overall, these findings indicate that extracellular ATP is capable of transactivating EGFR in intact epithelia, via P2Y_2_R activation and ligand shedding at the apical surface, representing an important response mechanism of intact epithelia to cell activation by mechanical, chemical or biological stimuli, resulting in induction of downstream stress or wound responses.

### ATP-dependent Activation of Src/ADAM17/EGFR are Mediated by DUOX1

**Figure 5:**
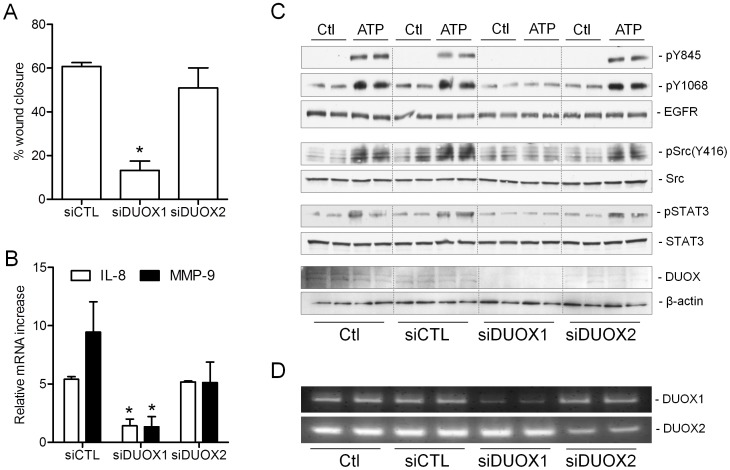
ATP-mediated wound responses and EGFR activation depend on DUOX1. (A) H292 cells were pre-incubated with DUOX1- or DUOX2-targeted siRNA or with control NS siRNA for 72 hrs, and subsequently serum-starved overnight and subjected to scratch wounding for analysis of wound closure after 8 hrs (A), stimulated with exogenous ATP (100 µM) for 8 hrs for analysis of ATP-dependent increases in mRNA expression of MMP-9 or IL-8 (B), or stimulated with ATP (100 µM) or EGF (100 ng/ml) for 10 min for analysis for phosphorylated and unphosphorylated forms of EGFR, c-Src, or STAT3 (C). Representative blots from 2–3 separate experiments are shown. Efficiency of siRNA-dependent silencing of DUOX1 or DUOX2 was evaluated by RT-PCR (D).

**Figure 6:**
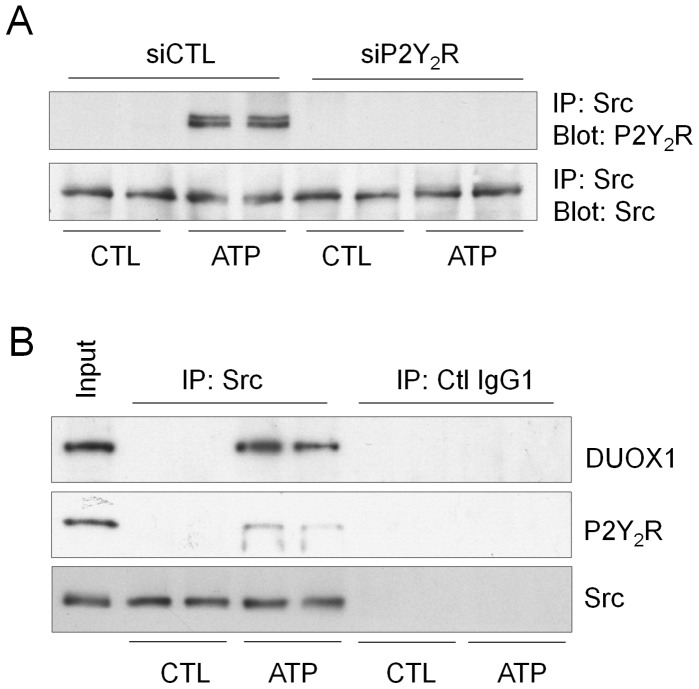
ATP stimulation promotes association between P2Y_2_R, Src and DUOX1. (A) H292 cells were pre-incubated with P2Y_2_R siRNA or control non-specific (NS) siRNA for 72 hrs, and stimulated with ATP (100 µM, 10 min), and Src was immunoprecipitated from cell lysates after which immunoprecipitated samples were evaluated for the presence of P2Y_2_R or Src by Western blot. (B) Untreated or ATP-treated H292 cells were immunoprecipitated with a-Scr or control IgG and immunoprecipitates were analyzed by Western blot for Src, P2Y_2_R, or DUOX1, in comparison with whole cell lysates (input). Representative blots of 2 experiments are shown.

Previous studies by us and others have suggested the importance of DUOX1 in epithelial wound responses and EGFR activation by various stimuli [10], [24], [36], although others have also suggested the importance of DUOX2 in epithelial responses to certain exogenous triggers [44]. We confirmed the importance of DUOX1 rather than DUOX2 in epithelial wound responses and in ATP-dependent induction of MMP-9 and IL-8 in H292 cells using siRNA silencing (**Fig. 5A,B**), which indicated that these EGFR-mediated outcomes are also dependent on DUOX1. Similarly, ATP-dependent phosphorylation of EGFR in H292 cells was found to depend on activation of DUOX1, whereas it was completely unaffected by silencing of DUOX2 (**Fig. 5C,D**). Moreover, silencing of DUOX1, but not DUOX2, also significantly attenuated ATP-dependent activation of Src, and also largely prevented tyrosine phosphorylation of the transcription factor STAT3, a downstream target of Src and/or EGFR involved in epithelial wound responses [45] (**Fig. 5C**). Silencing of DUOX1 also suppressed ATP-dependent production of H_2_O_2_, which was unaffected by DUOX2 siRNA, although the latter weakly suppressed basal H_2_O_2_ production (**Table 1**). Importantly, the effects of DUOX1 siRNA silencing could not be attributed to altered levels of Src or EGFR (**Fig. 6A**) or altered expression of P2Y receptors or ADAM17 (**Table SI**). Collectively, these findings indicate the critical importance of DUOX1-dependent H_2_O_2_ production in activation of Src/EGFR and subsequent wound responses.

**Table 1:** Contribution of DUOX1/2 to basal or ATP-dependent H_2_O_2_ production by H292 cells.

	H292	siCTL	siDUOX1	siDUOX2
Basal	0.014±0.002	0.020±0.004	0.022±0.002	0.007±0.003^a^
ATP	2.01±0.63	1.68±0.28	0.019±0.002^b^	1.76±0.30

H292 cells were transfected with indicated siRNA and cells were placed in HBSS and stimulated for 15 min with ATP (100 µM) after which conditioned media was mixed with tyrosine and LPO for HPLC analysis of dityrosine. Data are expressed as nmol H_2_O_2_/10^6^ cells and mean values SEM are presented from 4 measurements.

a p = 0.07 compared to siCTL.

b p<0.05 compared to siCTL.

Previous studies indicated that ATP-dependent activation of Src is facilitated by direct association of Src with P2Y_2_R at its SH3 binding domain [38]. Immunoprecipitation of Src from untreated or ATP-stimulated H292 cells confirmed a similar interaction between Src and P2Y_2_R in H292 cells in response to ATP stimulation (**Fig. 6A**). Furthermore, ATP stimulation also promoted a direct association between Src and/or P2Y_2_R with DUOX1 (**Fig. 6B**), further illustrating the potential involvement of DUOX1 in Src activation and downstream signaling in response to P2Y_2_R stimulation.

**Figure 7:**
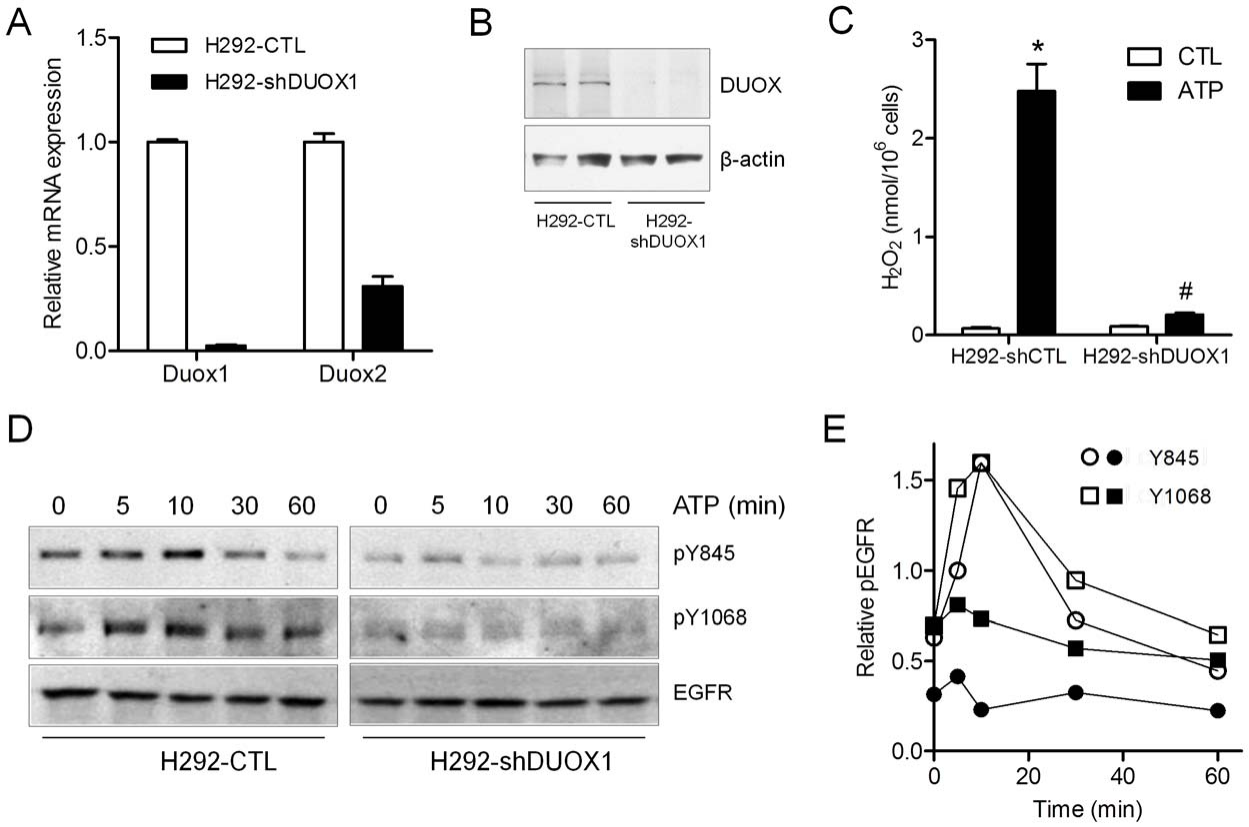
Lack of ATP-dependent EGFR phosphorylation in H292-shDUOX1 cells. Stably transfected H292-shDUOX1 or H292-CTL cells were evaluated for DUOX1 or DUOX2 RNA by qPCR (A), DUOX protein by western blot (B), or basal or ATP-stimulated H_2_O_2_ production (15 min), measured by HPLC analysis of peroxidase-catalyzed dityrosine (C). *: p<0.05 compared to untreated control; #:p<0.05 compared to H292-CTL (n = 6). Qualitatively similar findings were obtained using Amplex Red assay (not shown). Serum-starved H292-shDUOX1 or H292-CTL cells were stimulated with ATP for 5–60 min and harvested for western blot analysis for phosho-EGFR (Y845), phospho-EGFR (Y1068) and total EGFR (D), and results from 2–3 separate western blots were quantified (E).

To more definitively explore the importance of DUOX1 in EGFR transactivation by ATP and establish the DUOX1-dependent redox signaling mechanism(s), we generated H292 cell lines in which DUOX1 is stably and almost completely eliminated, by stable transfection with DUOX1-targeted shRNA (**Fig. S4**). Thus generated H292-shDUOX1 cells were found to almost completely lack DUOX1 mRNA and protein (**Fig. 7A,B**), and although H292-shDUOX1 cells produced similar extracellular H_2_O_2_ levels compared to control transfected cells (H292-shCTL), they failed to respond to exogenous ATP with respect to extracellular H_2_O_2_ production (**Fig. 7C**), indicating that they lack functional DUOX1. As expected, H292-shDUOX1 cells were resistant to ATP-induced EGFR phosphorylation at Y845 and Y1068 compared to corresponding control cells (H292-shCTL), although EGF-dependent EGFR phosphorylation was largely unaffected, and were also resistant to ATP- or EGF-dependent activation of Src, STAT3 or ADAM17 (**Fig. S5**). Kinetic analysis of ATP-induced EGFR activation indicated rapid phosphorylation at both Y845 and Y1068 in H292-shCTL cells (peaking at 5–10 min and returned to baseline after 30–60 min), whereas no significant increase in EGFR phosphorylation was observed at any time point in H292-shDUOX1 cells (**Fig. 7D,E**). These findings illustrate the importance of DUOX1 in initiation of EGFR phosphorylation by extracellular ATP, which contrasts with the recently reported regulation of EGFR by a different NADPH oxidase, NOX4, which was primarily found to regulate the duration of EGFR activation due to PTP1B inactivation without significantly affecting initial EGFR activation by EGF [**17**]. Collectively, our findings illustrate a critical role for DUOX1 in initiating EGFR transactivation by extracellular ATP, and possibly by other environmental stimuli, related to activation of Src and/or ADAM17.

### ATP-mediated DUOX1 Activation Results in Cysteine Oxidation within c-Src and ADAM17

**Figure 8:**
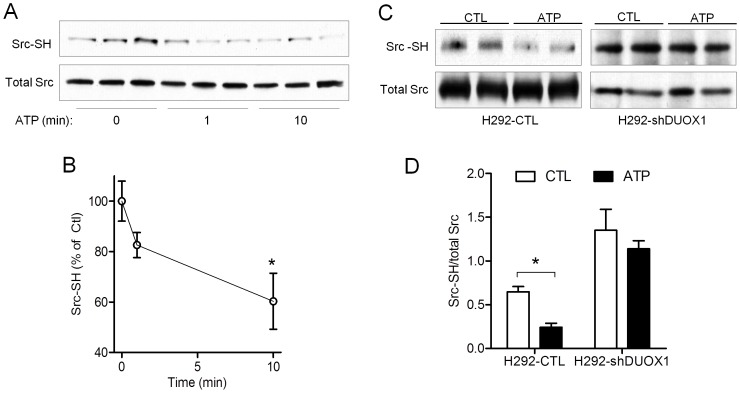
ATP stimulation induces DUOX1-dependent cysteine oxidation in Src. (A) Serum-starved H292 cells were stimulated with ATP (100 µM) for indicated times and lysed in the presence of Iodoacetyl-LC-Biotin, followed by purification of biotin-labelled protein with NeutrAvidin agarose, and western blot analysis of biotinylated proteins and whole lysates for Src, reflecting Src-SH and total Src, respectively. (B) Densitometry analysis of Src-SH status from 3 separate experiments. *p<0.05 compared to untreated control. (C) Similar analysis of Src-SH status in H292-shDUOX1 or corresponding H292-CTL cells after 10-min stimulation with ATP (100 µM). (D) Densitometry analysis of Src-SH relative to total Src based on Western blot analysis in panel C. Mean values of 4 replicates from 2 independent experiments are shown. *p<0.05 compared to untreated control.

While our results so far illustrate the importance of DUOX1 in activation of Src as a proximal mechanism in EGFR activation, the precise molecular mechanisms by which DUOX1-derived H_2_O_2_ mediates such response is not yet known. In this regard, various lines of evidence indicate that activation of Src is not only controlled by phosphorylation/dephosphorylation, but is also subject to redox control by reversible oxidation of critical cysteine residues [46]. Because of the reported role of P2Y_2_R in DUOX1 activation [47], and the direct association between P2Y_2_R, Src and DUOX in response to cell stimulation (**Fig. 6C**), we hypothesized that DUOX1-derived H_2_O_2_ might directly oxidize cysteine residues within Src and thereby promote its activity [31], [46]. To examine this, untreated or ATP-stimulated H292 cells were lysed in oxygen-free lysis buffer (under N_2_ atmosphere to minimize cysteine auto-oxidation) containing the thiol-specific biotin-labeling reagent, EZ-link iodoaceteyl-LC-Biotin (100 µM). Analysis of purified biotinylated proteins with an α-Src antibody demonstrated significant and time-dependent loss of biotinylated Src in response to ATP stimulation (**Fig. 8A,B**), reflecting loss of reduced cysteines within Src due to oxidation. Moreover, in comparison to ATP-dependent Src oxidation in H292-shCTL cells, no significant oxidation of Src was observed in ATP-stimulated H292-shDUOX1 cells (**Fig. 8C,D**), indicating the critical involvement of DUOX1 in Src oxidation. Based on previous findings showing that oxidation of selected cysteine residues within Src contributes to its full activation [**46**], our findings indicate that ATP-mediated DUOX1 activation promote Src activation by direct or indirect cysteine oxidation, and thereby promote downstream activation of ADAM17 and EGFR.

**Figure 9:**
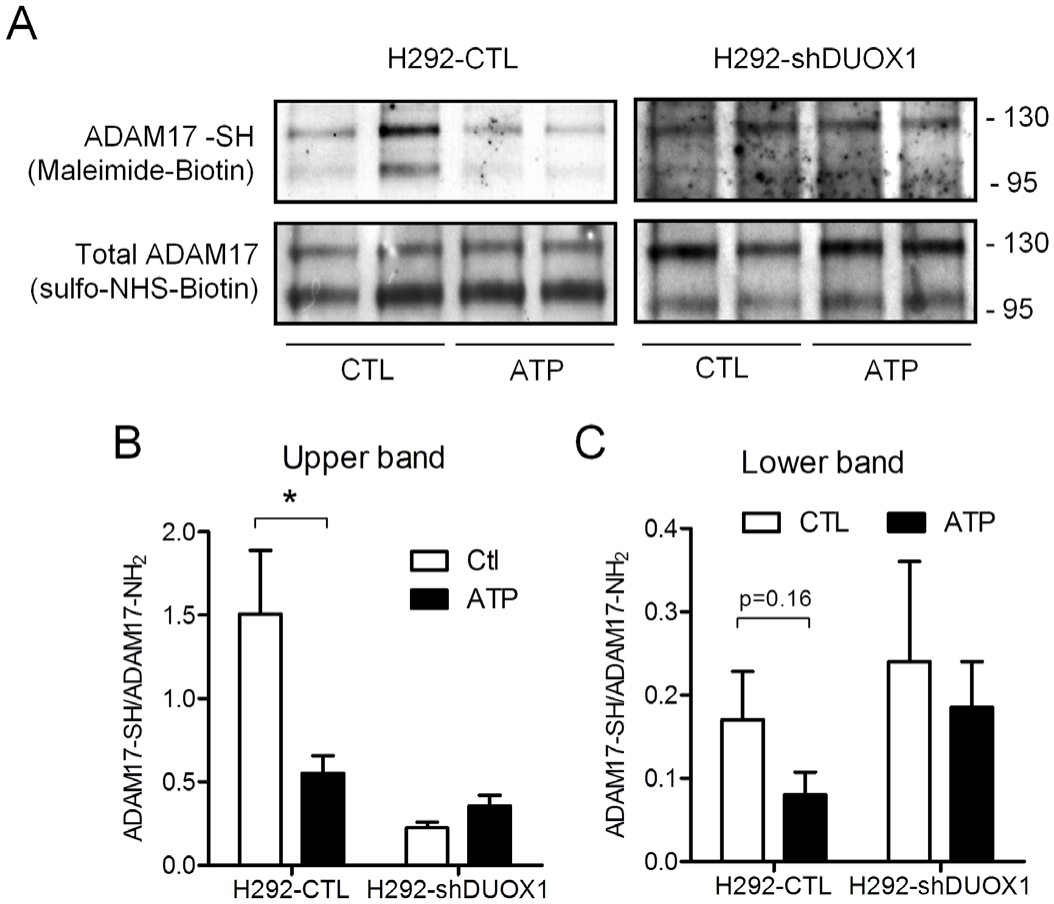
ATP stimulation induced DUOX1-dependent oxidation of ADAM-17 at the cell surface. (A) Confluent H292-shDUOX1 or corresponding H292-CTL cells were stimulated with ATP (10 min; 100 µM), and surface proteins were labeled with sulfo-NHS-biotin or MPB for labeling of surface NH_2_ groups or cysteine-SH residues, respectively. Avidin-purified biotinylated proteins were probed for ADAM-17 by western blot. Representative blot of 2 separate experiments are shown. Densitometry analysis of SH labeling relative to NH_2_ labeling of upper 120 kD bands (B) and lower 100 kD bands (C) are shown from 4 replicates from 2 independent experiments. *p<0.05 compared to untreated control.

Our studies indicate the critical importance of Src in ATP-dependent activation of ADAM17 and EGFR ligand shedding. Although the mechanisms involved in regulating ADAM17 are complex and not fully understood [6], [7], recent studies indicate the presence of redox-sensitive cysteines within the extracellular region of mature ADAM17, that appear to be subject to redox control regulate ADAM17 activity by switching from a “closed” inactive state to a more “open” active state at the cell surface [25], [26]. We considered a potential role of DUOX1 in similar redox regulation of cysteine residues within mature ADAM17, and used labeling with a cell-impermeable thiol-specific biotinylating agent, EZ-link Maleimide-Biotin (MBP), to determine the cysteine status within ADAM17 at the epithelial surface. As a loading control, cell-surface ADAM17 was also labeled with amino-specific biotinylation with sulfo-NHS-biotin, which is insensitive to H_2_O_2_. Biotinylated proteins were avidin-purified and probed by Western blot analysis of ADAM17, which indicated loss of thiol-specific biotinylation of ADAM-17 in response to ATP, as shown by reduced band intensity at 120 kD and 100 kDa, which represent proADAM17 and mature ADAM17, respectively (**Fig. 9**). Similar analysis of amino-labeled ADAM17 with sulfo-NHS-biotin did not show any change in response to ATP, illustrating selective oxidation of cysteine residues within ADAM17 following ATP stimulation. Moreover, whereas significant cysteine oxidation within ADAM17 was observed in H292-shCTL cells in response to ATP, no such alterations were seen in H292-shDUOX1 cells (**Fig. 9**), indicating the critical involvement of DUOX1. Collectively, these findings demonstrate that ATP-mediated activation of DUOX1 results in specific cysteine oxidation of both Src and ADAM17, thereby promoting their activity and subsequent activation of EGFR signaling.

## Discussion

**Figure 10:**
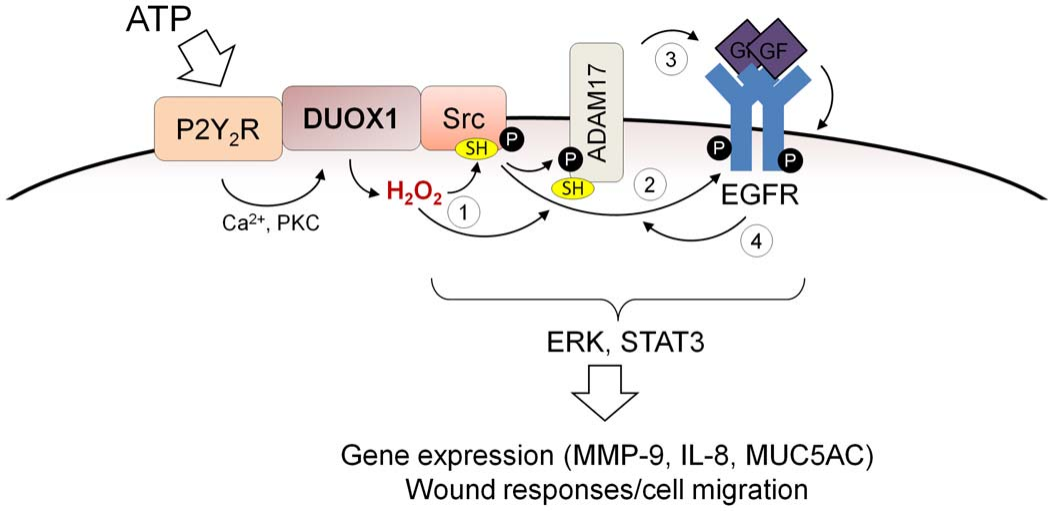
Schematic representation of proposed involvement of DUOX1 in ATP-mediated EGFR transactivation. ATP-mediated activation of P2Y_2_R promotes Ca^2+^ and protein kinase C (PKC) and thereby activates DUOX1-dependent H_2_O_2_ production. DUOX1-derived H_2_O_2_ promotes activation of Src and ADAM-17 by cysteine oxidation (1), and Src activation also promotes ADAM-17 activity by phosphorylation and directly activates EGFR by phosphorylation at Y845 (2). In addition, ADAM-17 activation promotes EGFR ligand shedding resulting in further EGFR activation (3). Activated EGFR can in turn promote activation of Src and ADAM-17 in a feed-forward mechanism to enhance EGFR signaling and downstream pathways such as ERK1/2 and STAT3 (4).

The present studies highlight the central involvement of the NADPH oxidase DUOX1 in EGFR transactivation in tracheobronchial epithelial cells in response to exogenous ATP, and thereby further establish DUOX1 as a critical factor in innate defense mechanisms within the respiratory epithelium. The effects of ATP are mediated by initial activation of P2Y_2_R purinergic receptors at the epithelial surface, which have been implicated in regulation of epithelial fluid and mucin secretion to promote mucociliary clearance [8], [12], wound responses that facilitate epithelial repair upon injury [10], [11], [13], and inflammatory cytokine production [24], [48]. Since many of these functional responses are also associated with activation of EGFR signaling [1], [2], [49], the interactions between P2Y_2_R and EGFR signaling form an area of much recent interest [38], [39], [50], [51]. Our findings demonstrate the importance of the non-receptor tyrosine kinase Src in ATP-dependent EGFR transactivation, consistent with findings that Src can directly interact with P2Y_2_R and EGFR to facilitate such signaling [30], [38], as well as a critical involvement of EGFR ligand shedding by ADAM17, consistent with earlier findings in other cell types [13]. Moreover, our present studies demonstrate the critical involvement of the NADPH oxidase DUOX1 which promotes EGFR transactivation by oxidative modification of critical cysteines within Src as well as ADAM17. Our collective findings regarding the proposed role of DUOX1 in ATP-mediated EGFR activation are schematically illustrated in **Fig. 10**.

The present studies shed additional light on the overall functional role of DUOX1 within the respiratory epithelium, which remains incompletely understood [52]. In addition to its proposed function as a host defense enzyme that provides a source of extracellular H_2_O_2_ that contributes to oxidative microbial killing within the airway lumen [53], [54], findings from the present study further establish DUOX1 as a mediator of redox signaling within the epithelium by promoting discreet and reversible oxidative modifications within specific target proteins to regulate their activity. Our present findings establish the importance of DUOX1 in activation of Src-EGFR and downstream wound responses as indicated by activation of the transcription factor STAT3 and induction of wound genes such as MMP-9 or IL-8 [1], [10], [37], [45]. Elegant studies in other organisms have demonstrated similar roles for DUOX in host and wound responses, highlighting the contribution of DUOX in specific signaling pathways that orchestrate these responses [55], [56], [57], suggesting that such functional properties of DUOX are highly conserved. One complicating factor in mammalian systems, however, is the presence of 2 separate DUOX isoforms rather than a single *DUOX* gene, with distinct modes of regulation and cellular localization [35], [58], [59], [60]. Indeed, while recent studies implicate DUOX2 in epithelial immune responses to TLR activating stimuli [44], [61], other reports suggest the importance of DUOX1 in epithelial wound responses, mucus production, and cytokine signaling [10], [36], [62], and our findings clearly establish that ATP-dependent EGFR activation is exclusively mediated by DUOX1 and not by DUOX2. These various findings may suggest variable interactions of specific DUOX isoforms with different GPCR or TLR. Indeed, our findings in **Fig. 6B** indicate that DUOX1 is directed associated with P2Y_2_R and Src in response to ATP stimulation, although we did not address whether this interaction was specific for DUOX1. The reported localization of Src [63] as well as DUOX1 [64] in lipid rafts in response to appropriate activation further supports such a direct interaction in membrane signaling complexes.

Although EGFR is primarily localized in the basolateral membrane within the intact epithelium and its full activation by ligand shedding, which occurs at the apical epithelial surface or within the intracellular space, is thought to require mechanical stress or disruption of epithelial junctions [42], [65], our studies indicate that luminal ATP was capable of activating EGFR in intact epithelial monolayers without the need for disruption of epithelial junctions, and disruption of tight junctions by EGTA-mediated Ca^2+^ chelation in fact diminished ATP responses. Such findings are consistent with the reported location of P2Y_2_R and DUOX at the apical epithelial surface, and suggest that Src, ADAM17, and EGFR must be also be partially localized at the apical epithelial surface to allow for such signaling. Alternatively, ligand-independent EGFR activation may be transduced through the cell by indirect EGFR-dependent ADAM-17 activation in a feed-forward mechanism [66].

Our findings extend our understanding of the role of H_2_O_2_ or ROS in regulation of EGFR signaling. Various previous findings have the demonstrated ability of ROS to activate EGFR and indicated an important role for Src in such activation [27], [28], whereas others have demonstrated ROS-dependent EGFR ligand shedding by activation of ADAM17 [24], [36]. Oxidative regulation of EGFR signaling may depend on whether EGFR activation occurs in the context of global oxidative stress or as part of a physiological signal, such as in GPRC-dependent EGFR activation. Indeed, oxidative stress has been demonstrated to result in aberrant EGFR activation and defective EGFR trafficking in relation to Src activation [28], [67]. In contrast, endogenous H_2_O_2_ production by NADPH oxidases appears to promote EGFR activation by patho-physiological stimuli, as a critical mechanism of physiological EGFR regulation [17], [36]. Our studies indicate the involvement of DUOX1-derived H_2_O_2_ in EGFR activation by concerted oxidative activation of both Src and ADAM17 in a pathophysiological setting of P2Y_2_R receptor activation by extracellular ATP, which is readily released from epithelial cells as a danger signal. The involvement of DUOX1 in EGFR transactivation also differs from the previously reported role of NOX4 in prolonging EGFR activation through inhibition of PTP1B [17], and occurs at the level of initial activation of EGFR signaling through Src and ADAM17.

Activation of Src appears to be central in ATP-mediated EGFR transactivation, since it can directly phosphorylate EGFR at Y845 [30], [68] and also contributes to activation of ADAM17 [41]. While the precise mechanism of Src activation by H_2_O_2_ is currently unknown, a number of reports indicate that Src and related kinases, such as Lyn and Yes, are subject to redox regulation which involves oxidation of one or more specific conserved cysteine residues within a cysteine-cluster in the C-terminal region [32], [33]. It has been suggested that tyrosine auto-phosphorylation at Y416 and cysteine oxidation within Src are successive events during e.g. cell adhesion and spreading, and that cysteine oxidation at C245 and C487 is necessary for full Src activation [46]. Our findings, however, indicate that Src phosphorylation at Y416 in response to ATP depends on DUOX1, whereas EGF-dependent phosphorylation of Src Y416 was more modest and appeared to be independent of DUOX1 (**Fig. S5**). These results suggest that DUOX1-dependent cysteine oxidation within Src also contributes to its auto-phosphorylation of Y416, and is required for its full activation, as was suggested previously [46]. However, it is important to point out that our studies did not elucidate the critical cysteine residues involved, and various Cys residues have been implicated in both positive and negative regulation of Src activity [31], [33], [69]. Further studies with selected Src mutants will be needed to more definitively address this. Also, our studies did not address the potential activation of other Src family kinases such as Fyn or Yes that may also be involved in innate epithelial responses to pollutants or infectious stimuli [34], [70].

ADAM17 plays a central role in the release of growth factors, cytokines, and other membrane proteins, and is rapidly activated by a variety of signaling pathways although the underlying mechanisms are still poorly understood [6]. Consistent with a recent report [41], our results demonstrate the critical importance of Src in ADAM17 activation by either ATP or EGF, the latter finding indicating that EGFR-dependent activation of ADAM17 may serve to augment or prolong EGFR activation in a feed-forward mechanism. In accordance with several recent reports [25], [26], we also provide evidence of cysteine oxidation with ADAM17 in response to ATP stimulation in a DUOX1-depedendent fashion. It was proposed that such cysteine oxidation within the mature form of ADAM17 at the cell surface alters its conformation to a more “open” state and promotes its activity, and that such oxidation occurs via thiol-disulfide exchange after initial oxidation of protein disulfide isomerase (PDI) [25]. We attempted to detect reduced or oxidized PDI at the cell surface of H292 cells, but were unsuccessful.

In summary, our findings highlight a central role of the NADPH oxidase DUOX1 in EGFR transactivation in airway epithelial cells in response to P2Y_2_R stimulation by exogenous ATP, and identify Src and ADAM17 as proximal targets for redox regulation in response to DUOX1 activation. While our findings provide further insight into the functional importance of DUOX1 in airway epithelial signaling and biology, our studies provoke several questions. First, our studies did not address how such DUOX1-dependent signaling is terminated, and future studies will be necessary whether such signaling is controlled by common reductases such as thioredoxins or glutaredoxins. Second, our studies did not establish the oxidation products within Src or ADAM17, or whether these enzymes are directly targeted by DUOX1-derived H_2_O_2_. In this regard, kinetic arguments suggest that H_2_O_2_-dependent protein cysteine oxidation is in most cases too slow compared to reactions with H_2_O_2_-metabolizing enzymes, and redox signaling by e.g. NADPH oxidase-derived H_2_O_2_ is thought to be largely mediated by a select number of sensitive H_2_O_2_ sensor proteins that can then transfer cysteine oxidation to less intrinsically susceptible proteins [71], [72]. Indeed, the peroxiredoxin (Prx) thiol peroxidases present strong candidates for such a H_2_O_2_ sensor role because of their cellular abundance and exquisite reactivity towards H_2_O_2_, and several findings indeed confirm the involvement of Prx in transducing oxidative signals towards various signaling proteins via thiol-disulfide exchange [71], [73]. It remains to be established whether Prx plays a similar role in P2Y_2_R-mediated oxidative activation of Src and ADAM17, or whether Src could possess similar redox sensor properties.

## Materials and Methods

### Cell Culture and Treatments

NCI-H292 cells, a human pulmonary mucoepidermoid carcinoma cell line, were originally obtained from the American Type Culture Collection (ATCC) and grown in RPMI 1640 medium containing 10% fetal bovine serum and 1% penicillin/streptomycin at 37°C and 5% CO_2_. For experimentation, cells were seeded in 24-well plates, grown to confluence and serum-starved for 24 hrs prior to treatments. Cells were placed in fresh serum-free medium for 2 hrs and stimulated with either exogenous ATP (Sigma, St. Louis, MO; 100 µM) or EGF (Calbiochem; 100 ng/ml), and cell lysates were collected at various time points by placing cells on ice in 100 µl lysis buffer (Cell Signaling, Beverly, MA) per well for 20 min. Lysates were collected by scraping, briefly sonicated, and cleared of insoluble material by centrifugation (14,000 rpm, 5 min) for analysis. In appropriate cases, inhibitors were added 30 min prior to cell stimulation.

Additional experiments were performed with immortalized human bronchial epithelial (HBE1) cells, originally provided by Drs. Yankaskas and Wu, which were cultured in Ham’s F-12/Dulbecco’s modified Eagle’s medium (50∶50) supplemented with insulin (5 µg/ml), transferrin (5 µg/ml), EGF (10 ng/ml), dexamethasone (0.1 µM), cholera toxin (10 ng/ml), and bovine serum albumin (0.5 mg/ml), and bovine hypothalamus extract (15 µg/ml). HBE1 cells were seeded on 12-well tissue culture inserts (Corning) coated with collagen (BD Biosciences; 50 µg/ml). After culture for 5 days to confluence, cells were grown at air-liquid interface conditions for an additional 14 days in the presence of 30 ng/ml retinoic acid to obtain polarized epithelia. One day prior to experimentation, cells were kept overnight in full media lacking EGF, and treatments with either ATP or EGF were added either apically (in 0.5 mL media) or basolaterally, and cells were collected as described above for analysis.

### Analysis of Epithelial Wound Responses

Confluent serum-starved H292 cells in 24-well plates were subjected to scratch injury using a sterile pipette tip, rinsed with warm PBS to remove cellular debris, and placed in fresh full media to monitor wound closure over 8 hrs. Initial and final wound areas were imaged using Image J software (NIH) for calculation of % wound closure. Alternatively, serum-starved H292 cells were stimulated with ATP (100 µM) for 8 hrs and RNA was extracted for analysis of mRNA levels of the common wound response genes MMP-9 and IL-8.

### Western Blot Analysis

Lysates containing equal amounts of protein (20–35 µg, measured using BCA protein assay kit; Pierce) were loaded on 10% SDS-PAGE gels and transferred to nitrocellulose membranes, and probed using antibodies against P2Y_2_R (Invitrogen; 1∶250), or EGFR (C74B9; 1∶1000), pEGFR Tyr845 (1∶500), pEGFR Tyr1068 (D7A5; 1∶1000), p-Src Tyr 416 (1∶1000), Src (L4A1; 1∶1000), p-STAT3 Tyr705 (D3A7; 1∶1000), STAT3 (79D7; 1∶1000), and β-actin (1∶1000; Cell Signaling). Primarily antibodies were probed with rabbit or mouse-specific secondary antibodies conjugated with HRP (Cell Signaling) and detected by enhanced chemiluminescence (Pierce).

### SiRNA Silencing of P2Y2R, Src, ADAM17, or DUOX1/2

H292 cells were seeded at 70% confluence for siRNA transfection in serum-free medium with siGENOME or ON-TARGET*plus* SMARTpool® siRNAs targeted against P2Y_2_R, Src, or ADAM17 or Non-Targeting siRNA Pool#1 as control (Dharmacon, Lafayette, CO) using DharmaFECT transfection reagent, according to the manufacturer’s instructions. Silencing of DUOX1 or DUOX2 was performed by transfection with the following siRNA reagents: DUOX1: (sense) GCUAUGCAGAUGGCGUGUAtt, (antisense) UACACGCCAUCUGCAUAGCtg; DUOX2: (sense) CGCAGUCAAUGUCUACAUCtt, (antisense) GAUGUAGACAUUGACUGCGtg, or with Silencer Negative Control #1 siRNA (Invitrogen). Following overnight transfection, medium was replaced with full RPMI 1640 medium (10% FBS & 1% pen/strep) the following day and cells were grown for 48 hrs until experimentation.

### Analysis of H_2_O_2_ Production

H292 cells were seeded at 150,000 cells/well in 24 plates and media was replaced with 200 µL HBSS for cell stimulation with ATP or EGF for 15 min, after which HBSS was removed and mixed with 10 µg/ml lactoperoxidase (Sigma) and 1 mM tyrosine for 15 min. Reactions were terminated by addition of 5% TCA for analysis of dityrosine production by HPLC [10]. Alternatively, H_2_O_2_ was analyzed using the Amplex Red assay (Invitrogen) according to the manufacturer’s instructions. H_2_O_2_ production was calculated using similar analysis of exogenous standards of H_2_O_2_ in HBSS, and expressed as nmol/10^6^ cells.

### Analysis of ADAM17 Activity

Confluent H292 cells were serum-starved and treated with ATP or EGF as described above, in the presence of a fluorogenic ADAM17 substrate (Calbiochem; 10 µM). Media was collected after 2 hrs, and ADAM17 activity was analyzed using a fluorescence plate reader. Inhibitors were added 30 min prior to stimulation with ATP or EGF.

### Generation of H292-shDUOX1 Cell Lines

H292 cells were transfected with pTET-On Advanced Vector (Clontech, Mountainview, CA) using Lipofectamine 2000 and transfected cells were selected using G418 (150 µg/ml). Selected H292-TetOn cells were subsequently transfected with pRNATin-H1.2/Hygro vector (GenScript, Piscataway, NJ) in which a short hairpin RNA sequence targeting DUOX1 was inserted, according to the manufacturer’s instructions (**Fig. S4**). Thus transfected H292-shDUOX1 cells were selected by culturing in the presence of hygromycin (1.5 µg/ml) for 2 weeks, and 6 colonies were picked and expanded. Corresponding control cell lines were generated by transfection of H292-TetOn cells with empty pRNATin-H1.2/Hygro vector (H292-CTL) and selected similarly. Initial analysis of selected H292-shDUOX1 cells showed a loss of DUOX protein expression after culture in the presence of doxycycline. However, upon further expansion of selected H292-shDUOX1 lines, levels of DUOX1 mRNA were found to be dramatically reduced compared to H292-CTL (**Fig. S4**), even in the absence of doxycycline, most likely due to known some basal expression associated with this 2^nd^ generation TetOn expression system (Clontech). Therefore, these cell lines were used in subsequent studies without addition of doxycycline.

### Immunoprecipitation of Src

Lysates of untreated or ATP-stimulated H292 cells containing 2 mg protein were pre-cleared with 50 µl protein A agarose beads (Santa Cruz) in a total volume of 1 ml for 15 mins at 4°C. Following brief centrifugation (3,000 rpm), supernatants were incubated with 2.5 µg of Src monoclonal mouse antibody (Cell Signaling) or equivalent amount of control IgG1, and incubated overnight at 4°C under continuous rotation, and subsequently mixed with protein A agarose beads (50 µl) for an additional 4 hrs at 4°C. Beads were washed 3X with lysis buffer and resuspended in 2X Laemmli’s sample buffer at boiled at 100°C for 10 min. Samples were loaded on 10% SDS-PAGE gels for Western blot analysis using rabbit antibodies against Src (L4A1, Cell Signaling), P2Y_2_R (1∶250; Invitrogen), or a polyclonal antibody against the Arg^618^–His^1044^ fragment of DUOX1 (kindly provided by Francoise Miot, Brussels, Belgium) [74].

### RT-PCR Analysis

Total RNA was extracted from cell lysates using the RNAeasy extraction kit according to the manufacturers protocol (Qiagen, Inc, Valencia, CA) and cDNA was synthesized using M-MLV reverse transcriptase and Oligo(dT)12–16 primer (Invitrogen). PCR amplifications were carried out for 30 cycles of denaturation (94°C, 1 min) annealing (58°C, 30 s), and extension (72°C, 1 min), using the following primer sets for DUOX1 (5′-GCA GGA CAT CAA CCC TGC ACT CTC-3′ and 5′-CTG CCA TCT ACC ACA CGG ATC TGC-3′) and DUOX2 (5′-GAT GGT GAC CGC TAC TGG TT-3′ and 5′-GCC ACC ACT CCA GAG AGA AG-3′), and the products were analyzed on 1% agarose gels. Expression of genes of interest was also analyzed by qPCR relative to GAPDH using SYBR Green PCR Supermix (BioRad) and the following with appropriate primers (Table S2) and normalized to GAPDH using the ΔΔC_T_ method.

### Detection of Thiol Status of Src and ADAM-17

Following treatments, NCI-H292 cells were lysed in deoxygenated lysis buffer (50 mM Tris-HCl, 150 mM NaCl, 0.5% (vol/vol) Triton X-100, 10 µg/ml of aprotinin and leupeptin), containing 100 µM EZ-link Iodoacetyl-LC-Biotin (Pierce), under N_2_ atmosphere to minimize artifactual cysteine oxidation during cell lysis [31]. Collected lysates were cleared by centrifugation (10 min, 14,000 rpm), and to remove excess iodoacetyl-LC-biotin, proteins were precipitated with 10% TCA and protein pellets were washed with cold acetone/HCl/H_2_O (at a ratio of 92∶2:10) and resuspended in lysis buffer containing 8 M urea. Samples containing equal protein concentrations (determined using TCA protein assay; Thermo Scientific) were mixed overnight with NeutrAvidin agarose beads (50 µl of 50/50 slurry; Pierce) at 4°C to collect biotinylated proteins. Beads were washed 3X with PBS to remove non-specific binding, and mixed with 2X reducing sample buffer containing β-mercaptoethanol and heated at 95–100°C to elute biotin-labeled protein from the resin. The amount of Src in these Biotinylated samples and initial lysates (input controls) were evaluated for Src content by SDS-PAGE and western blot analysis using a rabbit antibody against Src (Cell Signaling).

Thiol status of cell-surface ADAM-17 in treated H292 cells was analyzed essentially as described by Willems *et al*. [25]. Briefly, cells were incubated for 45 min with either EZ-Link sulfo-NHS (*N*-hydroxysuccinimido)-biotin (100 µM; Pierce) in PBS, or with 100 µM MPB [3-(*N*-maleimido-propionyl) biocytin; Pierce] in Hepes-buffered saline (21 mM Hepes, pH 7.05, 0.137 M NaCl, 5 mM KCl, 0.76 mM Na_2_HPO_4_·H_2_O and 5.56 mM dextrose), to label amino groups or thiol groups, respectively. Excess EZ-Link sulfo-NHS-biotin was quenched with 50 mM Tris/HCl (pH 7.5), and excess MPB was quenched with 200 µM GSH, after which cells were lysed in lysis buffer (50 mM Tris-HCl, 150 mM NaCl, 0.5% (vol/vol) Triton X-100, 10 µg/ml of aprotinin and leupeptin) for collection of biotinylated proteins as described above. Purified biotinylated proteins and original lysates were then analyzed by SDS-PAGE and western blotting using a rabbit anti-ADAM-17 antibody (Millipore; 1∶1000).

### Data Processing and Analysis

Western blots were semiquantified using densitometry analysis by ImageJ, and quantified data are presented as mean ± S.E. Statistical differences were analyzed using Student’s t-test and differences were considered significant at p<0.05.

## Supporting Information

Figure S1 **Pharmacological evaluation of P2Y-dependent EGFR activation.** Confluent serum-starved H292 cells were stimulated with either ADP, ATP, UDP, or UTP (100 µM each) for 10 min, cell lysates were analyzed for phosphorylated and unphosphorylated (total) EGFR by Western blot. Representative blots from 2 experiments are shown.
(TIF)

Figure S2 **ATP-dependent EGFR phosphorylation depends on Src.** Confluent serum-starved H292 cells were pre-treated with the EGFR kinase inhibitor AG1478 (10 µM; A) or with the Src inhibitor PP2 (1 µM; B) for 15 min and subsequently treated with ATP (100 µM) or EGF (100 ng/ml) for 10 min, unless indicated otherwise. Cell lysates were analyzed for phosphorylated EGFR by Western blot, and representative blots from 2–3 separate experiments are shown.
(TIF)

Figure S3 **Evaluation of ATP-dependent increase in ADAM17 activity.** Confluent H292 cells were pre-incubated with ADAM17 siRNA or control non-specific (NS) siRNA (Dharmacon) for 72 hrs, and ADAM17 mRNA expression was evaluated by qPRC (left) and ATP-stimulated (100 µM) activation of ADAM17 was determined using a fluorogenic substrate (right).
(TIF)

Figure S4 **Generation of stable H292-shDUOX1 cells.** (A) Generation of stable H292-shDUOX1 cells using pTet-On expression system (Clontech) and transfection with DUOX1-targeted shRNA using pRNATin-H1.2/Hygro vector (Genscript). (B) PCR analysis of DUOX1 or DUOX2 in H292-shDUOX1 cells and corresponding control cells (H292-CTL) grown in the absence or presence of doxycycline.
(TIF)

Figure S5 **ATP-dependent activation of EGFR, Src, or STAT3 is diminished in H292-shDUOX1 cells.** Confluent H292-shDUOX1 or corresponding H292-CTL cells were starved and stimulated with either ATP (100 µM) or EGF (100 ng/ml) for 10 min, and cell lysates were analyzed for phosphorylated and total EGFR, Src, or STAT3 (A). Western blots of phosphorylated forms of EGFR from 3 separate experiments were quantified by densitometry (B,C). ATP- or EGF-stimulated activation of ADAM17 in H292-shDUOX1 or H292-CTL cells assessed using fluorogenic ADAM17 substrate as in Fig. 4. (n = 3). *: p<0.05 compared to untreated cells; ^#^:p<0.05 compared with corresponding stimulation in H292-CTL cells.
(TIF)

Table S1 **Effects of DUOX siRNA silencing in H292 cells on mRNA expression of P2YR and ADAM17.**
(DOCX)

Table S2 **qPCR primer sequences used in this study.**
(DOCX)
