# Supplementary figures and images for: ATP-Mediated Transactivation of the Epidermal Growth Factor Receptor in Airway Epithelial Cells Involves DUOX1-Dependent Oxidation of Src and ADAM17
Source: PLoS One. 2013 Jan 18;8(1):e54391. doi: 10.1371/journal.pone.0054391 (PMC3548788; doi:10.1371/journal.pone.0054391)

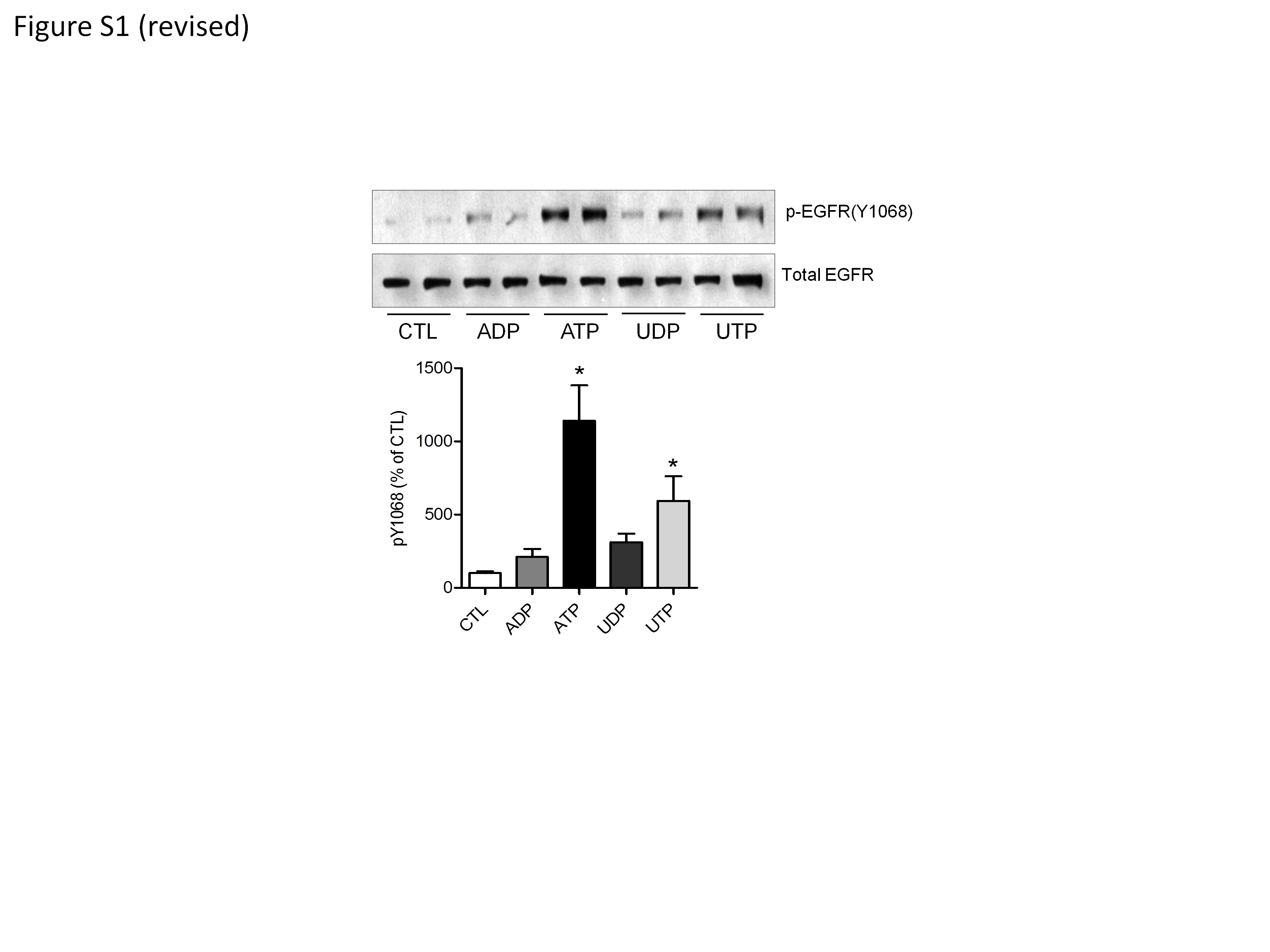

Supplement: Figure S1 — Pharmacological evaluation of P2Y-dependent EGFR activation. Confluent serum-starved H292 cells were stimulated with either ADP, ATP, UDP, or UTP (100 µM each) for 10 min, cell lysates were analyzed for phosphorylated and unphosphorylated (total) EGFR by Western blot. Representative blots from 2 experiments are shown. (TIF) [file pone.0054391.s001.tif]

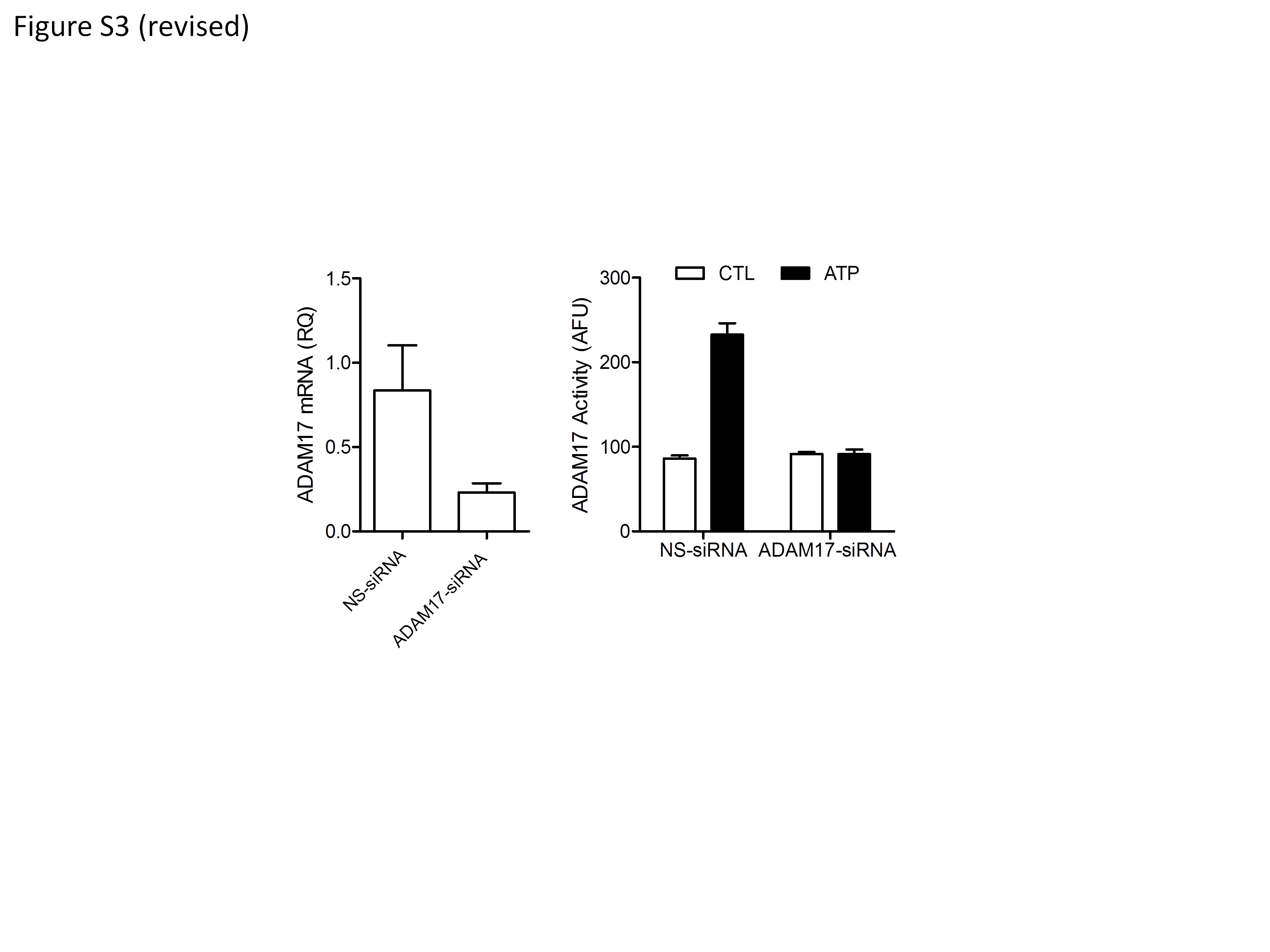

Supplement: Figure S3 — Evaluation of ATP-dependent increase in ADAM17 activity. Confluent H292 cells were pre-incubated with ADAM17 siRNA or control non-specific (NS) siRNA (Dharmacon) for 72 hrs, and ADAM17 mRNA expression was evaluated by qPRC (left) and ATP-stimulated (100 µM) activation of ADAM17 was determined using a fluorogenic substrate (right). (TIF) [file pone.0054391.s003.tif]

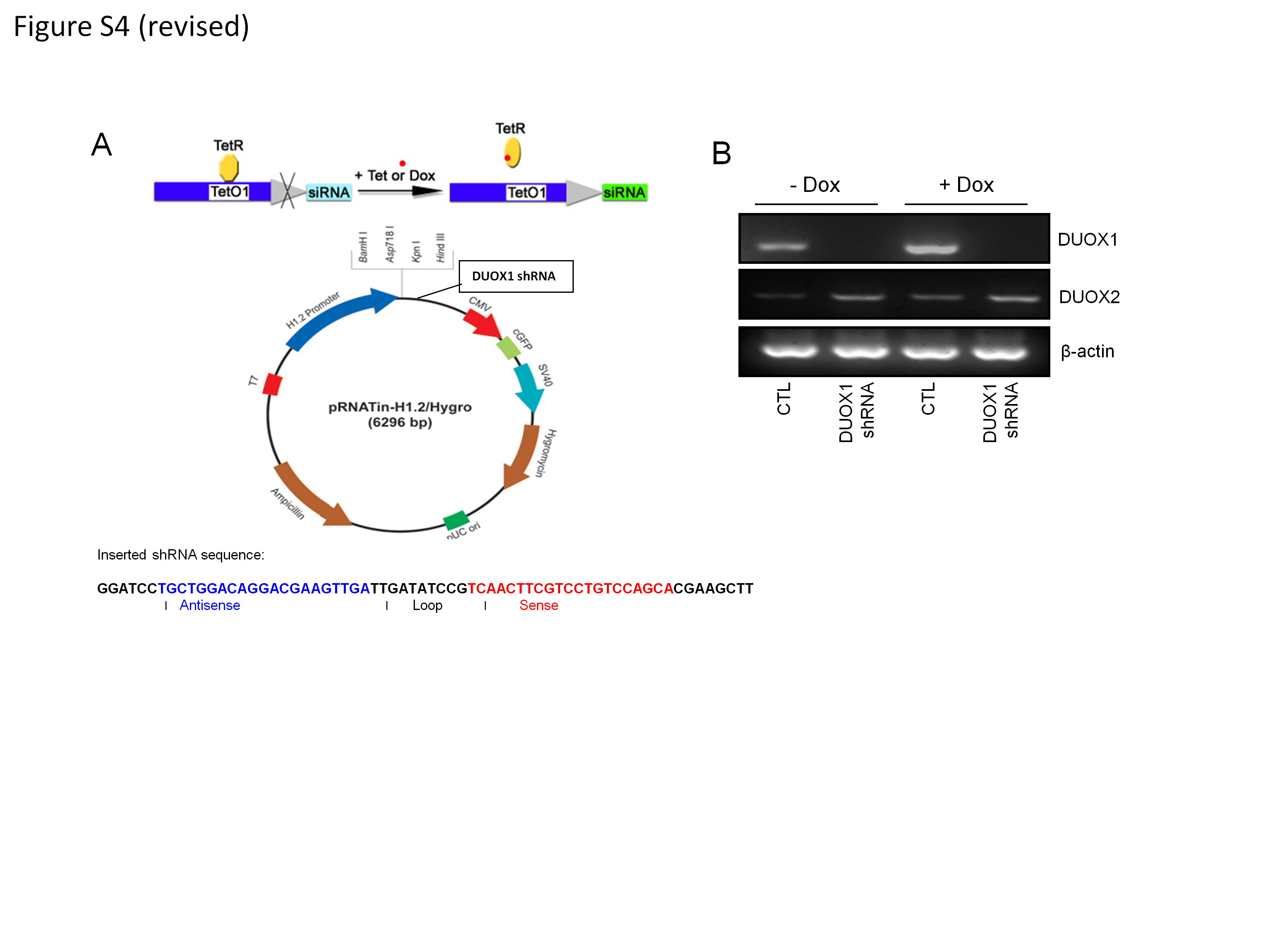

Supplement: Figure S4 — Generation of stable H292-shDUOX1 cells. (A) Generation of stable H292-shDUOX1 cells using pTet-On expression system (Clontech) and transfection with DUOX1-targeted shRNA using pRNATin-H1.2/Hygro vector (Genscript). (B) PCR analysis of DUOX1 or DUOX2 in H292-shDUOX1 cells and corresponding control cells (H292-CTL) grown in the absence or presence of doxycycline. (TIF) [file pone.0054391.s004.tif]

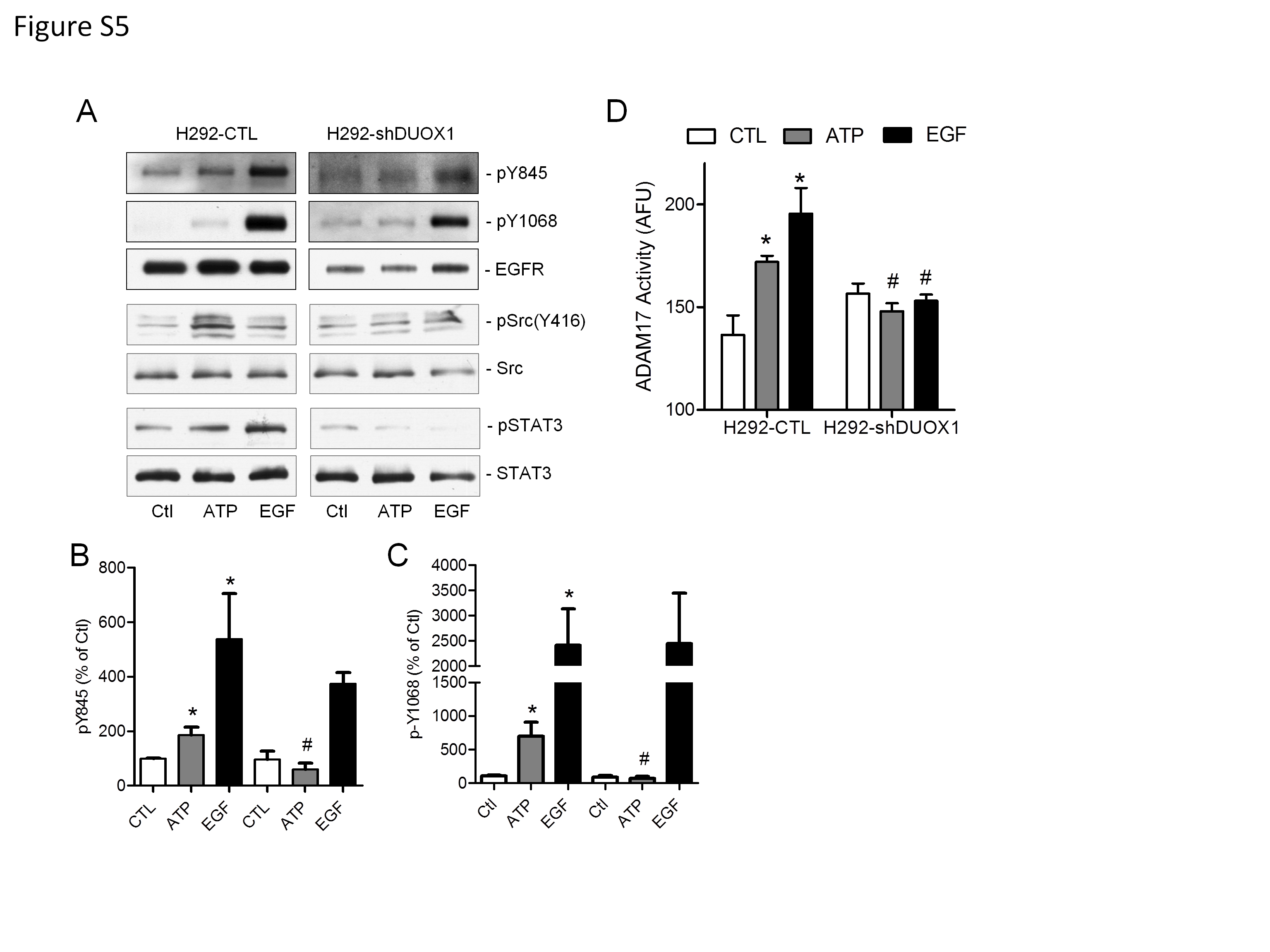

Supplement: Figure S5 — ATP-dependent activation of EGFR, Src, or STAT3 is diminished in H292-shDUOX1 cells. Confluent H292-shDUOX1 or corresponding H292-CTL cells were starved and stimulated with either ATP (100 µM) or EGF (100 ng/ml) for 10 min, and cell lysates were analyzed for phosphorylated and total EGFR, Src, or STAT3 (A). Western blots of phosphorylated forms of EGFR from 3 separate experiments were quantified by densitometry (B,C). ATP- or EGF-stimulated activation of ADAM17 in H292-shDUOX1 or H292-CTL cells assessed using fluorogenic ADAM17 substrate as in Fig. 4. (n = 3). *: p<0.05 compared to untreated cells; #:p<0.05 compared with corresponding stimulation in H292-CTL cells. (TIF) [file pone.0054391.s005.tif]
