# Supplementary material for: ATP-Mediated Transactivation of the Epidermal Growth Factor Receptor in Airway Epithelial Cells Involves DUOX1-Dependent Oxidation of Src and ADAM17
Source: PLoS One. 2013 Jan 18;8(1):e54391. doi: 10.1371/journal.pone.0054391 (PMC3548788; doi:10.1371/journal.pone.0054391)
Supplement: Table S1 — Effects of DUOX siRNA silencing in H292 cells on mRNA expression of P2YR and ADAM17. (DOCX) [file pone.0054391.s006.docx]

**Table S1**: Effects of DUOX siRNA silencing in H292 cells on mRNA expression of P2YR and ADAM17.

|  | siCTL | siDUOX1 | siDUOX2 |
| --- | --- | --- | --- |
| P2Y_1_R | 1.21 ± 0.23 | 0.68 ± 0.04 | 1.03 ± 0.20 |
| P2Y_2_R | 1.00 ± 0.06 | 1.42 ± 0.32 | 0.73 ± 0.36 |
| P2Y_4_R | 1.24 ± 0.10 | 1.29 ± 0.24 | 0.94 ± 0.11 |
| P2Y_6_R | 0.70 ± 0.15 | 0.40 ± 0.02 | 0.81 ± 0.05 |
| ADAM17 | 1.04 ± 0.06 | 1.42 ± 0.32 | 0.73 ± 0.36 |

H292 cells were transfected with indicated siRNA and mRNA expression of various P2YR and of ADAM17 was performed by qPCR using the Ct method. Results are expressed as relative RQ values normalized to expression in untransfected H292 cells. Mean values SE from 2-3 analyses are shown.
