# Supplementary material for: ATP-Mediated Transactivation of the Epidermal Growth Factor Receptor in Airway Epithelial Cells Involves DUOX1-Dependent Oxidation of Src and ADAM17
Source: PLoS One. 2013 Jan 18;8(1):e54391. doi: 10.1371/journal.pone.0054391 (PMC3548788; doi:10.1371/journal.pone.0054391)
Supplement: Table S2 — qPCR primer sequences used in this study. (DOCX) [file pone.0054391.s007.docx]

**Table S2: qPCR primer sequences used in this study**

| DUOX1 | F: ttcacgcagctctgtgtcaa, |
| --- | --- |
|  | R: agggacagatcatatcctggct |
| DUOX2 | F: acgcagctctgtgtcaaaggt, |
|  | R: tgatgaacgagactcgacagc |
| P2RY1 | **F**: **ctgtgcaatgccttaggact** |
|  | **R**: **tacttggaggctatgccttg** |
| P2RY2 | **F: gccagtgtgaggctgtaact** |
|  | **R: agccaactggctttacagtg** |
| P2RY4 | **F**: **atcacccgcaccatttacta** |
|  | **R:** **atatttgtccccagtgagca** |
| P2RY6 | **F**: **gtctaccgcgagaacttcaa** |
|  | **R**: **tgatcaccttgggcatagtt** |
| ADAM-17 | **F**: **tacagatagagcagattcgcattct** |
|  | **R**: **ctctagcaacatcttcacatccc** |
| MMP-9 | F: ctctgg aggttcgacgtg |
|  | R: gtc cac ctg gtt caa ctc ac |
| IL-8 | F: tagcaaaattgaggccaagg |
|  | R: agcagactagggttgccaga |
| GAPDH | F: gaaggctggggctcatttg |
|  | R: aggctgttgtcatacttctcatgg |
